# Dexamethasone Alleviates Myocardial Injury in a Rat Model of Acute Myocardial Infarction Supported by Venoarterial Extracorporeal Membrane Oxygenation

**DOI:** 10.3389/fpubh.2022.900751

**Published:** 2022-07-19

**Authors:** Xingdong Cheng, Rongzhi Zhang, Shilin Wei, Jian Huang, Kerong Zhai, Yongnan Li, Bingren Gao

**Affiliations:** ^1^Department of Cardiac Surgery, Lanzhou University Second Hospital, Lanzhou University, Lanzhou, China; ^2^Department of Anesthesiology, Lanzhou University Second Hospital, Lanzhou University, Lanzhou, China

**Keywords:** dexamethasone, venoarterial extracorporeal membrane oxygenation, acute myocardial infarction, inflammatory response, apoptosis

## Abstract

Myocardial ischemia causes myocardial inflammation. Research indicates that the venoarterial extracorporeal membrane oxygenation (VA ECMO) provides cardiac support; however, the inflammatory response caused by myocardial ischemia remains unresolved. Dexamethasone (Dex), a broad anti-inflammatory agent, exhibits a cardioprotective effect. This study aims to investigate the effect of Dex on a rat model of acute myocardial infarction (AMI) supported by VA ECMO. Male Sprague-Dawley rats (300–350 g) were randomly divided into three groups: Sham group (*n* = 5), ECMO group (*n* = 6), and ECMO + Dex group (*n* = 6). AMI was induced by ligating the left anterior descending (LAD) coronary artery. Sham group only thoracotomy was performed but LAD was not ligated. The ECMO and ECMO + Dex groups were subjected to 1 h of AMI and 2 h of VA ECMO. In the ECMO + Dex group, Dex (0.2 mg/kg) was intravenously injected into the rats after 1 h of AMI. Lastly, myocardial tissue and blood samples were harvested for further evaluation. The ECMO + Dex group significantly reduced infarct size and levels of cTnI, cTnT, and CK-MB. Apoptotic cells and the expression levels of Bax, Caspase3, and Cle-Caspase3 proteins were markedly lower in the ECMO + Dex group than that in the ECMO group. Neutrophil and macrophage infiltration was lower in the ECMO + Dex group than in the ECMO group. A significant reduction was noted in ICAM-1, C5a, MMP-9, IL-1β, IL-6, and TNF-α. In summary, our findings revealed that Dex alleviates myocardial injury in a rat model of AMI supported by VA ECMO.

## Introduction

Ischemic heart disease (IHD) is the leading cause of morbidity and mortality across the globe, accounting for 9 million deaths annually (1, 2). Based on the American Heart Association, the current incidence of IHD is estimated at 18.2 million cases in the United States (2). Among many types of IHD, acute myocardial infarction (AMI) is the most prevalent. Although early reperfusion is the current effective treatment strategy (3) that improves the overall prognosis, AMI remains a significant cause of cardiogenic shock (CS), with a high mortality rate of up to 40–50% in the last two decades (4–6). Because of ischemic injury, numerous damage-associated molecular patterns (DAMPs) are released during the early stages of AMI (7–10), which in turn triggers an intense inflammatory response to remove necrotic debris. Furthermore, several pro-inflammatory cytokines and chemokines are released causing an inflammatory process that digests damaged cells and extracellular matrix (ECM) tissue, hence enlarged infarcted size. Therefore, it is important to understand the mechanisms of solving inflammation of myocardial necrosis.

Venoarterial extracorporeal membrane oxygenation (VA ECMO) is considered a rescue strategy, providing temporary circulatory and respiratory support, and has been extensively used as a salvage therapy for patients with CS (11–13). Nonetheless, myocardial inflammation persisted during VA ECMO remained unresolved. This limits the benefits VA ECMO can bring to patients.

Dex is popular for its promising anti-inflammatory and immunosuppressive effects (14) and widely applied in the clinical treatment of inflammatory and autoimmune diseases (15, 16). Moreover, studies indicate that Dex influences the heart through various mechanisms. Libby and co-workers reported that pharmacologic doses of Dex reduced myocardial infarction size. Elsewhere, Spath et al. discovered that treatment with Dex prevents the loss of myocardial lysosomal and stabilizes endothelial cell membranes (17). Additionally, Dex preconditioning has a protective impact on myocardial ischemia-reperfusion injury in rats (18). Nonetheless, it remains unclear whether Dex can optimize VA ECMO causing better clinical outcomes among high-risk patients with CS after AMI.

Based on the above studies, we proposed the following hypothesis: Dex alleviates myocardial injury in a rat model of AMI supported by VA ECMO through anti-inflammatory effects.

## Materials and Methods

### Animals

**Figure 1:**
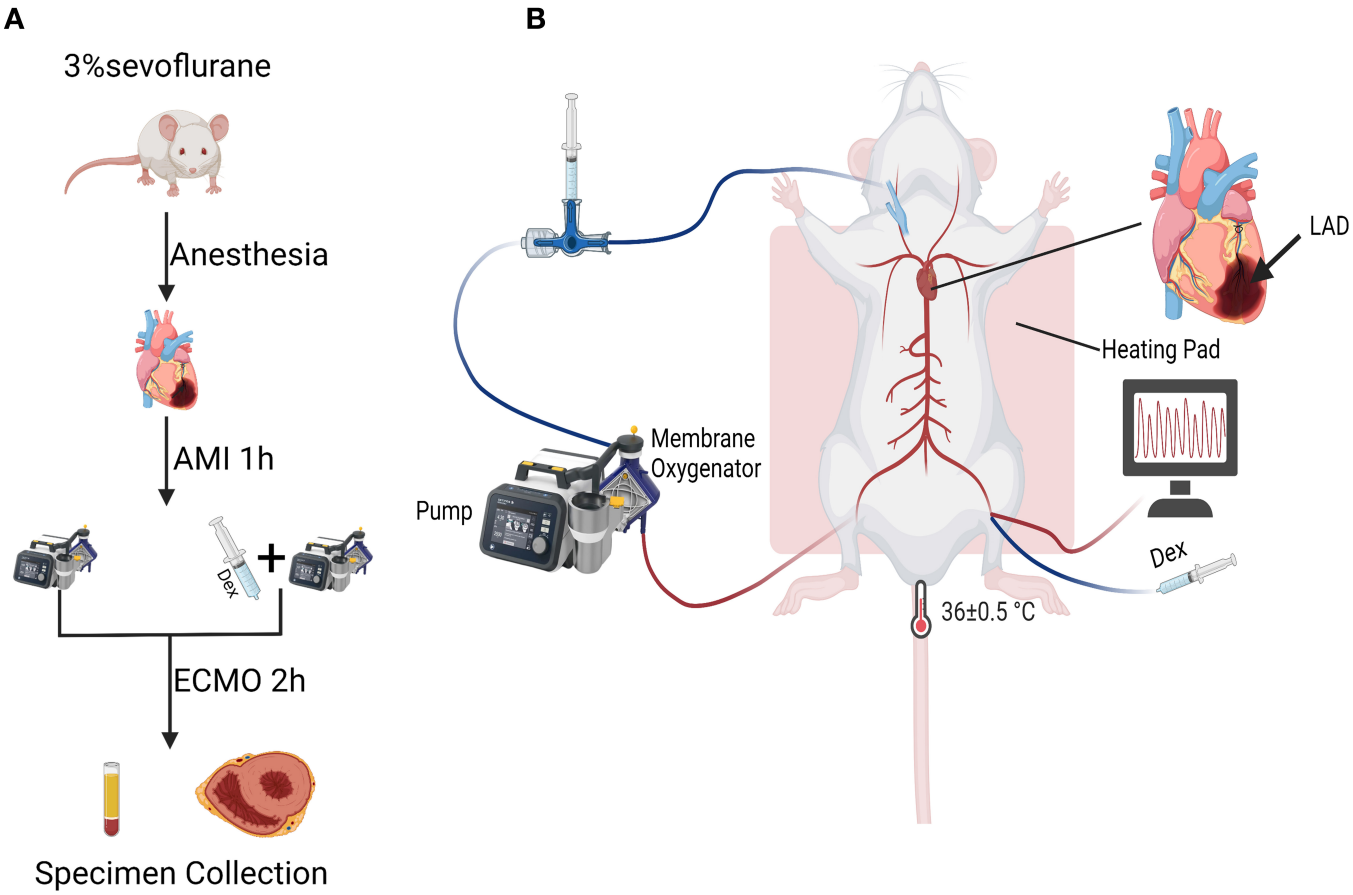
Flow chart and schematic diagram of experimental operation. **(A)** Flow chart of the establishment of experimental models. **(B)** Schematic diagram of the application of Dex in a rat model of AMI supported by VA ECMO.

A total of 17 Sprague-Dawley rats (300 to 350 g) were purchased from the Veterinary Institute, Chinese Academy of Agricultural Sciences (Lanzhou, China) and randomly divided into three groups: Sham group (*n* = 5), ECMO group (*n* = 6) and ECMO + Dex group (*n* = 6). Figure 1A shows flow chart of the establishment of experimental models. Figure 1B demonstrates schematic diagram of the application of Dex in a rat model of AMI supported by VA ECMO. All experimental protocols were approved by the local Ethical Committee of Lanzhou Second Hospital, Lanzhou University, Gansu (No. P2020-18), and were performed in compliance with “Guide for the care and use of laboratory animals” published by the US National Institutes of Health (NIH Publication No. 85-23, revised 1996).

### Anesthesia and Surgical Preparation

Rats were anesthetized using 3% sevoflurane, followed by oral endotracheal intubation using a 16-gauge catheter and mechanically ventilated under a respiratory frequency of 70–75 breaths/min, a tidal volume of 6 mL/kg, and an I:E ratio of 1:2 using a mini-ventilator (Alcott Biotech, China). The rats were placed in a supine position on a heating pad and the rectal probe was inserted to continuously monitor central temperature and maintained at 36 ± 0.5°C. After exposing the left femoral artery, it was cannulated using a 24-gauge catheter to continuously monitor the mean arterial pressure (MAP) and obtain blood samples throughout the procedure.

### Acute Myocardial Infarction Modeling

All operations were conducted under general anesthesia. The heart was exposed by a left thoracotomy. Subsequently, AMI was induced by ligating the proximal LAD using a 5-0 prolene suture in the ECMO and ECMO + Dex groups. The successful performance of LAD ligation was confirmed by the pale coloring of the infarcted left ventricular wall.

### Venoarterial Extracorporeal Membrane Oxygenation Modeling

VA ECMO circuit system designed for the rats comprised a peristaltic pump (PreFluid, China), a membrane oxygenator and tubing (Xijing Medical, China), a specially designed venous drainage tube, a 22-gauge and a 24-gauge catheter (Wego, China). The required prime volume for the oxygenator and the circuit tubing was 8 mL, including 6% hydroxyethyl starch (HES) (3 mL) and sodium lactate Ringer (3 mL). First, a 22-gauge catheter was inserted in the right femoral artery as the arterial perfusion end of the VA ECMO. Subsequently, a specially designed venous drainage tube was inserted in the right jugular vein as the venous drainage end of VA ECMO. Heparin (300 U/kg) was administered in each rat. After 1 h of LAD ligation, adjuvant therapy was performed by initiating VA ECMO at a flow rate of 80–90 mL/kg/min according to blood pressure to reach optimal flow rate in the ECMO and the ECMO + Dex group. Additionally, Dex (0.2 mg/kg) was injected into the left femoral vein of rats 1 h after ligation of LAD in the ECMO + Dex group. After reaching an optimal flow rate, it was adjusted to a level that can maintain desired arterial pressure. Notably, MAP was recorded throughout the process. Gas flow (90% O_2_) was initiated at about 80–100 mL/min and adjusted according to FiO_2_ and sweep rate to maintain blood gasses within the physiological range. After 2 h of VA ECMO support, the rats were weaned off VA ECMO, and the remaining priming volume was re-infused. After the procedure, rats were euthanized using pentobarbital overdoses administered intravenously. Subsequently, heart tissue and blood samples of rats were collected for further experiments.

### Blood Gas Analysis

Arterial samples for blood gas analysis were obtained through the left femoral artery at 0 (T_0_, baseline), 1 h (T_1_, after ligation of LAD), and 3.5 h (T_2_, after VA ECMO support). Then, arterial blood gas was measured using a blood gas analysis instrument (EG7+, iStar, Abbott, USA).

### Histopathology Evaluation

Heart tissues samples were harvested and stained with hematoxylin-eosin (H&E) and immunohistochemistry (IHC) according to the manufacturer's instructions. For the H&E staining, the number of cardiac constrictor zone necrosis was counted using ImageJ 8.4 software (National Institutes of Health, USA). For the IHC staining, the sections were overnight incubated at 4°C with the primary antibodies anti-Myeloperoxidase (MPO) (1:2,000, Abcam, USA) and anti-CD68 (0.5 ug/mL, Proteintech, China). The positive area of each image was analyzed using GraphPad Prism 8.0 (GraphPad, USA).

### 2,3,5-Triphenyltetrazolium Chloride (TTC) Staining

The hearts were harvested and rapidly frozen at −20°C for 15 min. Subsequently, the frozen heart was sectioned into five-axis slices (2 mm) from the apex to the bottom. The sections were incubated at 37°C in 2% TTC solution (Solarbio, China) for 18 min. Thereafter, the heart sections were washed three times with PBS solution and photographed. The myocardial infarction area (white) was analyzed using ImageJ 8.4 software (National Institutes of Health, USA).

### Enzyme-Linked Immunosorbent Assay (ELISA)

The contents of myocardial injury markers cTnI, cTnT, CK-MB in serum were detected using ELISA kits (Enzyme-linked Biotech, China) according to the manufacturer's instructions. Also, the levels of chemokines intercellular adhesion molecule-1β (ICAM-1), C5a, matrix metalloproteinase-9 (MMP-9), and IL-1β, IL-6, TNF-α in heart tissues were also detected using ELISA.

### Terminal-Deoxynucleotidyl Transferase-Mediated Nick end Labeling (TUNEL)

The number of apoptotic cells in paraffin sections was detected using the TUNEL assay (Roche, USA) according to the manufacturer's instructions. Five visual areas from each sample were randomly selected and analyzed using confocal microscopy (Carl Zeiss, Germany). Tunel positive rate was expressed as the ratio of positively stained nuclei to total nuclei using the ImageJ 8.4 software (National Institutes of Health, USA).

### Western Blot (WB)

Total proteins from the myocardial tissues were extracted using radioimmunoprecipitation (RIPA) lysis buffer (Beyotime, China) containing protease and phosphatase inhibitors for 30 min. Bicinchoninic acid assay (BCA, Solarbio, China) was used to quantify protein concentrations. Equal amounts of proteins from each sample were electrophoresed in sodium dodecyl sulfate-polyacrylamide gel electrophoresis, transferred to 0.45 μm polyvinylidene difluoride (PVDF) membranes and blocked with 5% defatted milk at room temperature for 1 h. Then, the blots were overnight incubated at 4°C with the indicated primary antibodies anti-Bcl-2 (1:2,000, Proteintech, China), anti-Bax (1:4,000, Abcam, USA), anti-Caspase3 (1:1,000, Proteintech, China), anti-Cleaved Caspase3 (1:2,000, CST, USA). The β-actin (1:5,000, Proteintech, China) was used as the reference gene for sample comparisons. After 1 day, PVDF membranes were incubated with horseradish peroxidase (HRP)-conjugated secondary antibodies (1:10,000, Proteintech, China) at room temperature for 1 h. Eventually, the enhanced chemiluminescence detection reagents (Millipore, USA) were used to scan PDVF membranes. Intensities of specific molecular bands were analyzed using the ImageJ 8.4 software (National Institutes of Health, USA).

### Statistical Analysis

All data were expressed as mean ± standard deviation (SD). Statistical analysis for different groups comparison was performed using the Student's *t*-test. *P* < 0.05 were considered statistically significant. All analyses were performed using the SPSS 22.0 (SPSS Software, USA) and GraphPad Prism 8.0 (GraphPad, USA).

## Results

### Perioperative Hemodynamics and Metabolic Parameters

**Figure 2:**
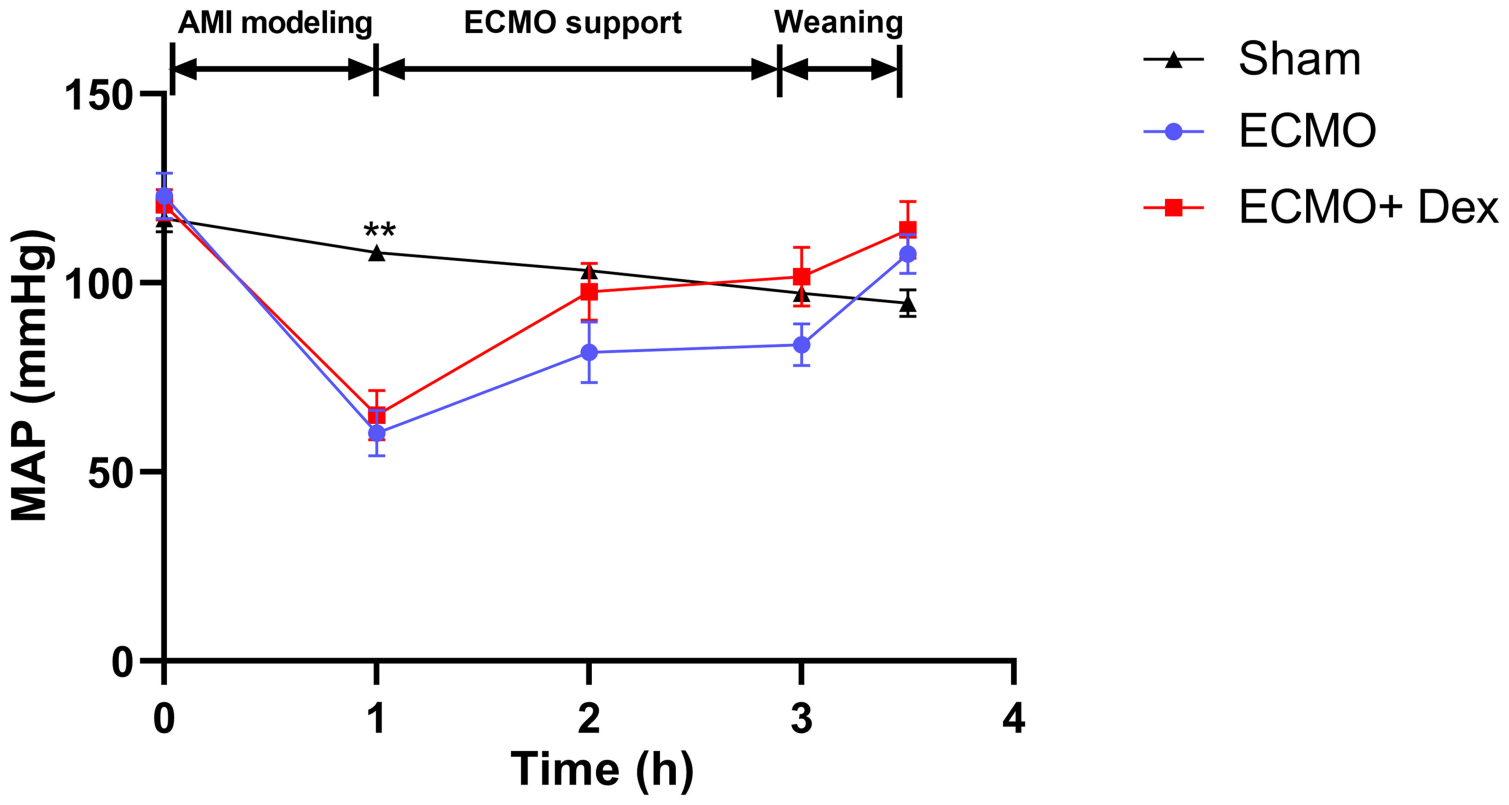
Dynamic changes of mean arterial pressure (MAP) in each group throughout the operation. **p < 0.01 vs. the ECMO group.

Figure 2 shows dynamic changes of MAP. Unlike before AMI modeling, MAP was significantly decreased after ligation of LAD but gradually increased and remained stable after VA ECMO support. Furthermore, MAP was higher in the ECMO + Dex group than that in the ECMO group and increased briefly, then returned to baseline levels after weaning off VA ECMO. Table 1 summarizes blood gas analysis parameters throughout the experiment. In contrast with the baseline, PaO_2_ and SaO_2_ were reduced after AMI modeling (*p* < 0.05). Hct and Hb were significantly decreased after VA ECMO support (*p* < 0.05). This may be attributed to blood dilution. Other parameters remained stable throughout the experiment.

**Table 1:** Biochemical parameters pre and post VA ECMO.

**Biochemical parameters**	**T_**0**_**	**T_**1**_**	**T_**2**_**
PH	7.324 ± 0.127	7.398 ± 0.141	7.466 ± 0.097
PaCO_2_ (mmHg)	39 ± 9	47 ± 5	43 ± 7
PaO_2_ (mmHg)	171 ± 19	130 ± 17^*^	234 ± 71
BE	0 ± 2	−1 ± 4	−2 ± 3
HCO${}_{3}^{-}$ (mmol/l)	24.9 ± 3.6	24.8 ± 1.8	24.4 ± 3.7
TCO_2_ (mmol/l)	23.2 ± 6.6	24.9 ± 3.2	25.6 ± 4.2
SaO_2_ (%)	99.6 ± 0.4	97.2 ± 1.1^*^	99.2 ± 0.5
Na^+^ (mmol/l)	142 ± 1	141 ± 4	139 ± 2
K^+^ (mmol/l)	4.5 ± 0.7	4.8 ± 0.3	4.9 ± 0.4
Ca^2+^ (mmol/l)	1.35 ± 0.07	1.27 ± 0.13	1.25 ± 0.09
Hct (%)	41.6 ± 1.7	40.8 ± 2.3	28.9 ± 2.3^#^
Hb (g/dL)	14.2 ± 0.3	14.4 ± 1.3	12.4 ± 1.1^#^

* *p < 0.05, T_1_ vs. T_0_;*

# *p < 0.05, T_2_ vs. T_1_*.

### Myocardial Injury

**Figure 3:**
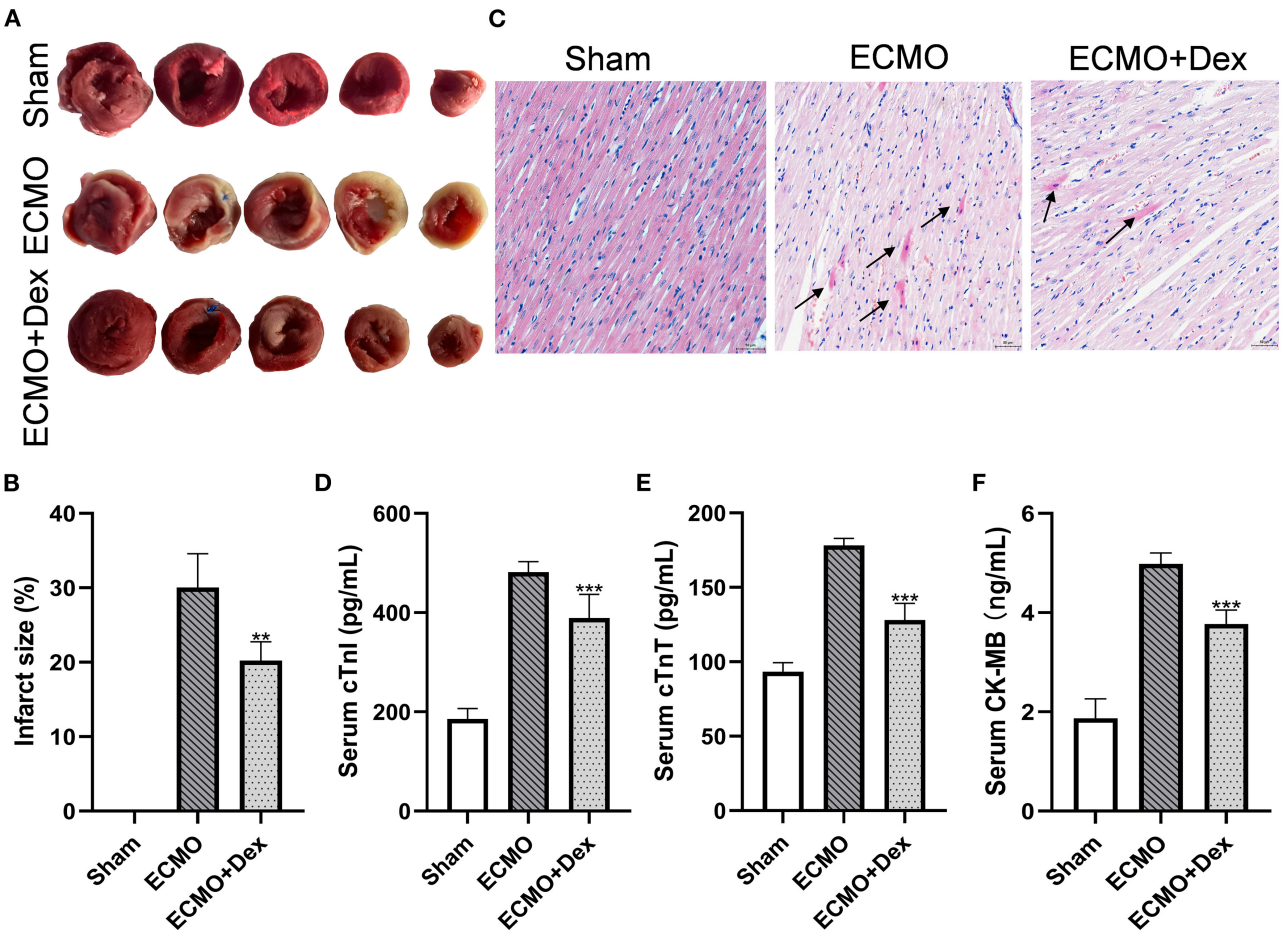
Dex treatment attenuated myocardial injury. **(A,B)** Representative TTC staining images and quantitative analysis in each group. **(C)** Representative H&E staining images in each group. Scale bar: 50 μm. Arrows indicate contraction band necrosis. **(D–F)** The concentrations of cardiac injury markers cTnI, cTnT, and CK-MB in the serum. Data are expressed as mean ± *SD*. **p < 0.01, ***p < 0.001 vs. the ECMO group.

Figure 3A demonstrates TTC staining for heart tissue. Myocardial infarct size was significantly increased in the ECMO group relative to the Sham group. However, we found infarct size have significantly reduced in the ECMO + Dex group than that in the ECMO group (*p* < 0.01; Figure 3B). Histological analysis revealed that the degree of contraction band necrosis was remarkably higher in the ECMO group compared with the Sham group. Nevertheless, this pathological change was significantly ameliorated in the ECMO + Dex group (Figure 3C). The levels of myocardial injury markers cTnI, cTnT, and CK-MB in the serum were significantly increased in the ECMO group relative to the Sham group. Unlike in the ECMO group, these findings were significantly reduced in the ECMO + Dex group (*p* < 0.001, *p* < 0.001, and *p* < 0.001, respectively; Figures 3D–F).

### Myocardial Apoptosis

**Figure 4:**
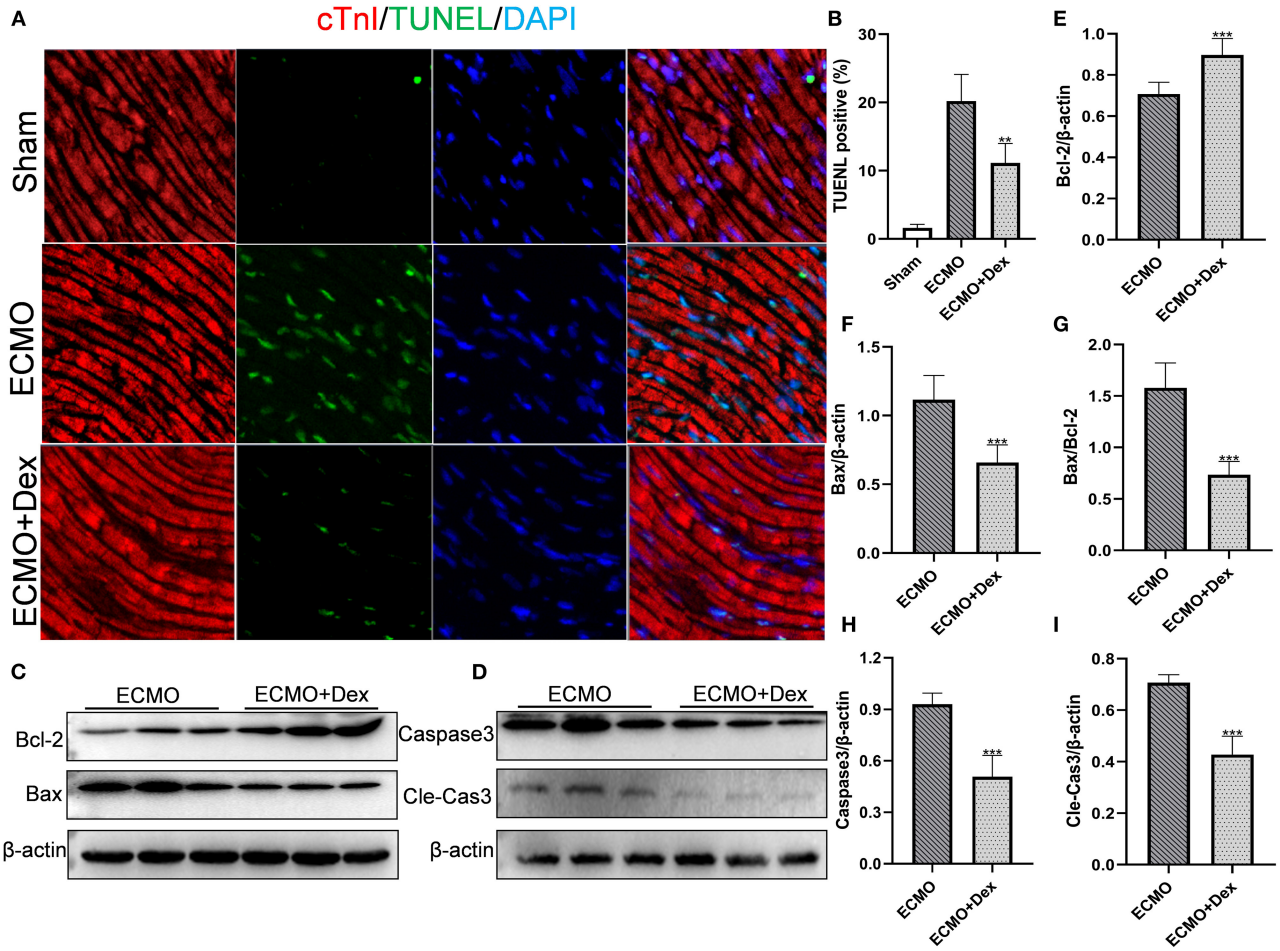
Dex treatment reduced myocardial apoptosis. **(A,B)** Representative TUNEL staining images and quantitative data in each group. **(C,D)** Representative images of Western Blot analysis in each group. **(E–I)** Relative expression levels of Bcl-2, Bax, Caspase3, and Cleaved Caspase3 in each group. Data are expressed as mean ± *SD*. **p < 0.01, ***p < 0.001 vs. the ECMO group.

The apoptotic rates for cardiac tissue were measured through Tunel staining (Figure 4A). The results showed that the number of apoptotic cells was markedly increased in the ECMO group compared with the Sham group. Apoptotic cells were significantly lower in the ECMO + Dex group relative to the ECMO group (*p* < 0.01; Figure 4B). Expression levels of apoptotic-related proteins were detected through Western blot (Figures 4C,D). Consequently, expression levels of Bax, the ratio of Bax/Bcl-2, Caspase3, and Cleaved Caspase3 were significantly downregulated in the ECMO + Dex group compared to that in the ECMO group (*p* < 0.001, *p* < 0.001, *p* < 0.001, and *p* < 0.001, respectively; Figures 4F–I). Nevertheless, Dex treatment significantly upregulated anti-apoptotic Bcl-2 expression compared to that in the ECMO group (*p* < 0.001; Figure 4E).

### Neutrophil and Macrophage Infiltration

**Figure 5:**
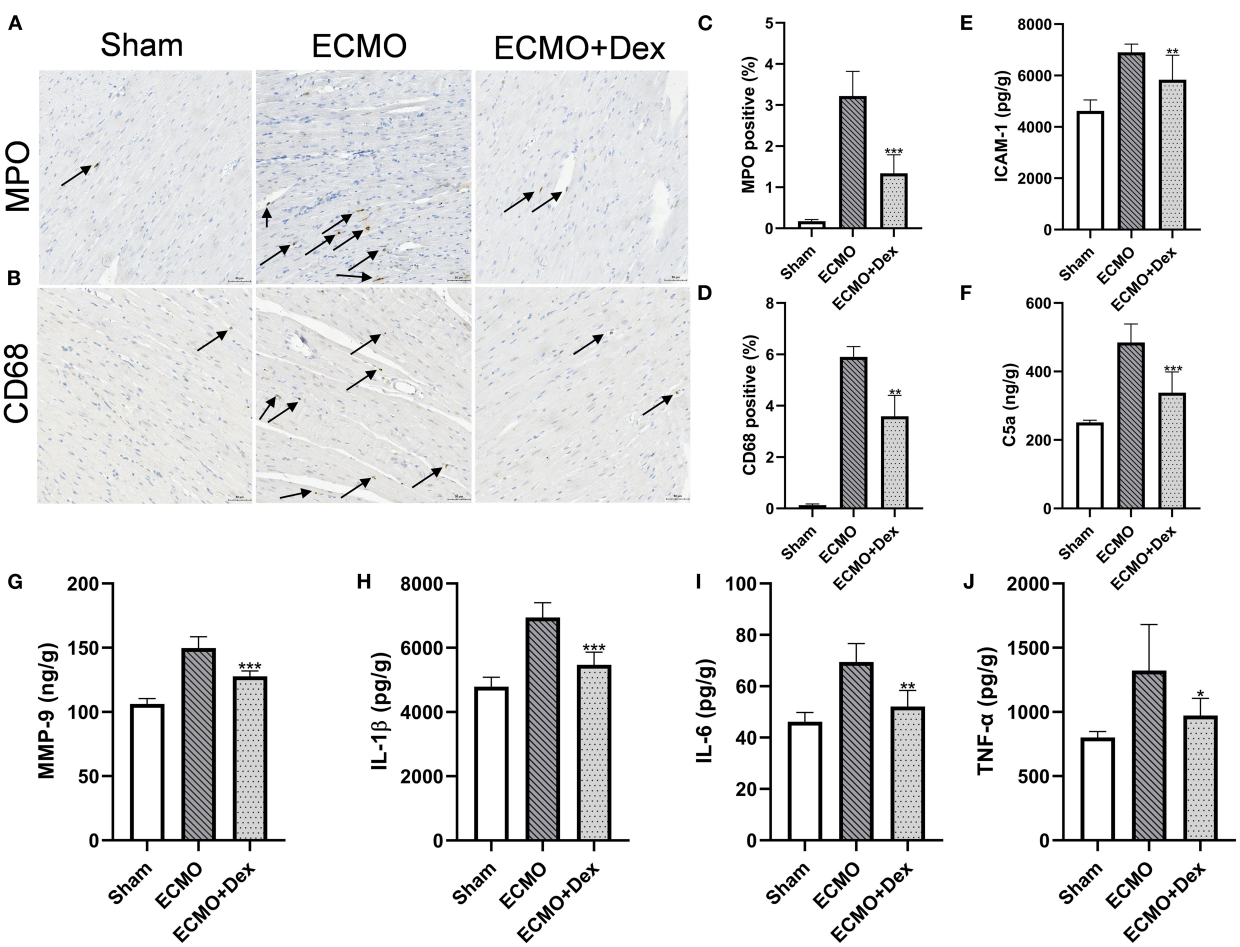
Dex treatment reduced chemotaxis effect and inflammatory response. **(A–D)** Representative images and quantitative data of neutrophils and macrophagocyte infiltrations. Scale bar: 50 μm. Arrows indicate neutrophils and macrophagocyte infiltrations. **(E–G)** The levels of chemokines ICAM-1, C5a, and MMP-9 for heart tissues in each group. **(H–J)** The levels of inflammatory factors IL-1β, IL-6, and TNF-α for heart tissues in each group. Data are expressed as mean ± SD. *p < 0.05, **p < 0.01, ***p < 0.001 vs. the ECMO group.

Figures 5A,B illustrate the effects of Dex on the number of neutrophil and macrophage infiltrations. The results showed that the number of neutrophil and macrophage infiltrations was markedly increased in the ECMO group relative to the Sham group. However, neutrophil and macrophagocyte infiltrations were significantly lower after Dex treatment (*p* < 0.001 and *p* < 0.01, respectively; Figures 5C,D).

### Chemokines and Inflammatory Factors

ELISA results revealed that the concentrations of chemokines factors ICAM-1, C5a, and MMP-9 were lower in the ECMO + Dex group than in the ECMO group (*p* < 0.01, *p* < 0.001, and *p* < 0.001, respectively; Figures 5E–G). In addition, concentrations of inflammatory factors IL-1β, IL-6, and TNF-α in the heart tissue were remarkably reduced in the ECMO + Dex group compared with the ECMO group (*p* < 0.001, *p* < 0.01, and *p* < 0.05, respectively; Figures 5H–J).

## Discussion

Several clinical studies indicate that VA ECMO potentially improves the survival rate in patients with CS after AMI. Besides, its early application triggers significant benefits (19, 20). However, significantly reducing myocardial injury under the VA ECMO support remains unsolved. Herein, we found that Dex significantly reduced myocardial infarct size, pathological myocardial damage, and the release of myocardial injury markers in a rat model of AMI supported by VA ECMO, this indicates that Dex could significantly enhance the effect of VA ECMO treatment.

Previous studies indicate that AMI induces a strong local and systemic inflammatory response, in turn, a complete inflammatory response often aggravates the tissue damage, leading to ventricular remodeling, dysfunction, and heart failure (21). Elsewhere, one study reported that the early phase aseptic inflammatory response of AMI regulates the progression of myocardial necrosis, ventricular remodeling, and heart failure after AMI (22). This causes an up-regulation of chemokines and cytokines as well as additional cell necrosis. Therefore, there is an urgent need for novel therapeutic methods that modulate the immune and inflammatory response after AMI based on molecular and cellular levels.

As a synthetic glucocorticoid (23), Dex regulates inflammatory and immune response by targeting cellular homeostasis and the gene activity and has been extensively used to treat critically ill patients due to its confirmed safety (24). Reports show that short-term treatment with Dex has a protective effect on myocardial ischemia and reperfusion injury (25). Therefore, Dex is a potential drug that reduces myocardial injury and enhances myocardial protection. Herein, using the rat model of AMI supported by VA ECMO, Dex significantly reduced the inflammatory response. Thus, the mechanism of Dex in alleviating myocardial injury may be exerted by inhibiting the inflammatory response during the process.

In the process of myocardial injury, many chemokines are released, then neutrophils, and macrophages are rapidly recruited to the site of injury or infection; notably, the neutrophils are usually considered first responders in the process (26, 27). MPO and CD68 are the primary surface markers of neutrophils and macrophages, respectively (28). IHC staining showed that Dex significantly reduced the infiltration of two types of inflammatory cells, and various chemokines primarily mediated the infiltration of inflammatory cells. Measurement results of chemokine levels in myocardial tissue revealed that the levels of chemokines ICAM-1, C5a, and MMP-9 were significantly decreased in the ECMO + Dex group compared to that in the ECMO group, indicating that Dex reduced the infiltration of inflammatory cells in myocardial tissue by suppressing the release of chemokines. We measured the levels of three types of inflammatory mediators including IL-1β, IL-6, and TNF-α in the myocardial tissue to further investigate whether Dex reduces inflammation response. IL-1β and IL-6 have been demonstrated as proinflammatory cytokines and were associated with myocardial necrosis (29, 30). TNF-α was one of the most important proinflammatory mediators produced by activated macrophages (31, 32). A considerable body of experimental evidence indicates that the above three inflammatory mediators regulate the inflammatory response (33). The detection of inflammatory mediators revealed that the number of inflammatory mediators was significantly lower in the ECMO + Dex group than that in the ECMO group. This suggests that Dex hinders inflammation response by reducing the release of inflammatory mediators.

Besides its anti-inflammatory effects, studies have also shown that Dex exhibits an anti-apoptosis effect. Furthermore, Xu et al. demonstrated that Dex potentially protects ischemic cardiomyocytes from apoptosis in myocardial infarction mice via transcriptional activation of the *Bcl-xL* gene (34). Our study further confirmed that Dex could significantly inhibit myocardial cell apoptosis in a rat model of AMI supported by VA ECMO.

This study has worth-mentioning limitations. VA ECMO support was performed for only 2 h to alleviate blood damage from the pump. Thus, additional studies are necessary to evaluate whether extended VA ECMO support will achieve better outcomes. Besides, Dex and ECMO were administered 1 h after LAD ligation in the study. Therefore, different time nodes will be set to further study the protective effect of Dex.

## Conclusions

In general, administration of Dex while initiating VA ECMO further alleviated myocardial injury by suppressing the inflammatory response and apoptosis in a rat model of AMI supported by VA ECMO. Thus, the treatment may be a new therapeutic strategy in the future.

## Data Availability Statement

The original contributions presented in the study are included in the article/supplementary material, further inquiries can be directed to the corresponding author/s.

## Ethics Statement

The animal study was reviewed and approved by the Animal Ethics Committee of Lanzhou University Second Hospital.

## Author Contributions

BG and YL conceived and designed the experiment. XC, RZ, SW, JH, and KZ carried out the experiment and analyzed the experimental data. XC retrieved the relevant literature and wrote the manuscript. BG provided helpful comments and revised the manuscript. All authors have read and approved the submitted version.

## Funding

This research was funded by the Talent Introduction Plan of the Lanzhou University Second Hospital (No. YJRCKYQDJ-2021-02), the Natural Sciences Foundation of Gansu (No. 20JR10RA760 and 20JR10RA745), the Scientific Research Projects of Colleges in Gansu Province (No. 2020B-028 and 2020B-049), and the Cuiying Scientific and Technological Innovation Program of Lanzhou University Second Hospital (No. CY2019-QN01 and CY2019-BJ07).

## Conflict of Interest

The authors declare that the research was conducted in the absence of any commercial or financial relationships that could be construed as a potential conflict of interest. The reviewer QW declared a shared affiliation with the author(s) to the handling editor at the time of review.

## Publisher's Note

All claims expressed in this article are solely those of the authors and do not necessarily represent those of their affiliated organizations, or those of the publisher, the editors and the reviewers. Any product that may be evaluated in this article, or claim that may be made by its manufacturer, is not guaranteed or endorsed by the publisher.
